# A Data-Driven Algorithm Integrating Clinical and Laboratory Features for the Diagnosis and Prognosis of Necrotizing Enterocolitis

**DOI:** 10.1371/journal.pone.0089860

**Published:** 2014-02-28

**Authors:** Jun Ji, Xuefeng B. Ling, Yingzhen Zhao, Zhongkai Hu, Xiaolin Zheng, Zhening Xu, Qiaojun Wen, Zachary J. Kastenberg, Ping Li, Fizan Abdullah, Mary L. Brandt, Richard A. Ehrenkranz, Mary Catherine Harris, Timothy C. Lee, B. Joyce Simpson, Corinna Bowers, R. Lawrence Moss, Karl G. Sylvester

**Affiliations:** 1 State Key Laboratory of Industrial Control Technology, Institute of Industrial Process Control, Zhejiang University, Hangzhou, Zhejiang, China; 2 School of Health Management, Hangzhou Normal University, Hangzhou, Zhejiang, China; 3 Department of Surgery, Stanford University, Stanford, California, United States of America; 4 College of Computer Science and Technology, Zhejiang University, Hangzhou, Zhejiang, China; 5 Department of Surgery, Johns Hopkins University School of Medicine, Baltimore, Maryland, United States of America; 6 Department of Surgery, Texas Children’s Hospital, Baylor College of Medicine, Houston, Texas, United States of America; 7 Department of Pediatrics, Yale University School of Medicine, New Haven, Connecticut, United States of America; 8 Department of Pediatrics, Children’s Hospital of Philadelphia, Philadelphia, Pennsylvania, United States of America; 9 Division of Pediatric Surgery, Nationwide Children’s Hospital, Columbus, Ohio, United States of America; 10 Department of Surgery, Ohio State College of Medicine, Columbus, Ohio, United States of America; Emory University School of Medicine, United States of America

## Abstract

**Background:**

Necrotizing enterocolitis (NEC) is a major source of neonatal morbidity and mortality. Since there is no specific diagnostic test or risk of progression model available for NEC, the diagnosis and outcome prediction of NEC is made on clinical grounds. The objective in this study was to develop and validate new NEC scoring systems for automated staging and prognostic forecasting.

**Study design:**

A six-center consortium of university based pediatric teaching hospitals prospectively collected data on infants under suspicion of having NEC over a 7-year period. A database comprised of 520 infants was utilized to develop the NEC diagnostic and prognostic models by dividing the entire dataset into training and testing cohorts of demographically matched subjects. Developed on the training cohort and validated on the blind testing cohort, our multivariate analyses led to NEC scoring metrics integrating clinical data.

**Results:**

Machine learning using clinical and laboratory results at the time of clinical presentation led to two NEC models: (1) an automated diagnostic classification scheme; (2) a dynamic prognostic method for risk-stratifying patients into low, intermediate and high NEC scores to determine the risk for disease progression. We submit that dynamic risk stratification of infants with NEC will assist clinicians in determining the need for additional diagnostic testing and guide potential therapies in a dynamic manner.

**Algorithm availability:**

http://translationalmedicine.stanford.edu/cgi-bin/NEC/index.pl and smartphone application upon request.

## Introduction

Necrotizing enterocolitis (NEC) is one of the most common life-threatening diseases of the newborn and predominantly affects low birth weight infants in the first weeks of life with a reported frequency of between 1% and 5% of NICU admissions [1], [2]. Published data from the NICHD neonatal network reports that 7% of very low birth-weight (VLBW < 1,000 gm.) infants develop definitive NEC (Bell’s stage II) [3]. The pathogenesis of NEC includes progressive inflammation of the newborn gut involving enteric bacteria, the innate immune system and a compromised intestinal epithelial barrier resulting in eventual necrosis in advanced cases. In general, NEC can be thought of as occurring in two forms, medical (non-progressive) and surgical NEC (progressive). The extent of inflammation and intestinal injury in medical NEC is limited and reversible. In surgical NEC, the inflammatory process progresses resulting in irreversible necrosis and gangrene thus necessitating surgical resection of the affected segments. Published overall mortality rates for NEC range from 15% to 30%, and are higher in those infants requiring surgical intervention for progressive cases [4], [5]. Several studies have demonstrated that the need for surgical intervention for NEC is an independent risk factor for long-term growth abnormalities, adverse neurodevelopmental outcomes and gastrointestinal morbidity including short bowel syndrome [5], [6].

Bell’s stage [7] is currently the most commonly utilized set of clinical criteria for diagnosing NEC. Bell’s staging criteria are however susceptible to inter-observer subjectivity and overall do not forecast course of disease. In general, Bell’s staging criteria are utilized to classify neonates under consideration of NEC as being suspected, confirmed and advanced. The determination of confirmed disease normally requires radiographic confirmation thus signifying intramural intestinal gas as a result of enteric bacterial invasion. The identification of neonates with progressive disease by clinical parameters alone generally occurs after a time when meaningful changes in patient management could significantly alter the course of disease. Taken together, these studies highlight the increasing biomedical burden imposed by NEC and therefore the need for better risk-stratification models of NEC in order to assist in altering its onset and progression.

We postulated that NEC-associated clinical and laboratory data patterns could improve both the diagnostic accuracy and the clinical outcome prediction of infants under suspicion of having NEC. We utilized statistical learning methods to develop and validate NEC diagnostic and prognostic algorithms. We also propose the application of existing and evolving information technologies to facilitate adoption and deliver point-of-care application of our novel NEC predictive algorithm (Figure S1).

## Materials and Methods

### Subjects and associated clinical data sets

**Figure 1:**
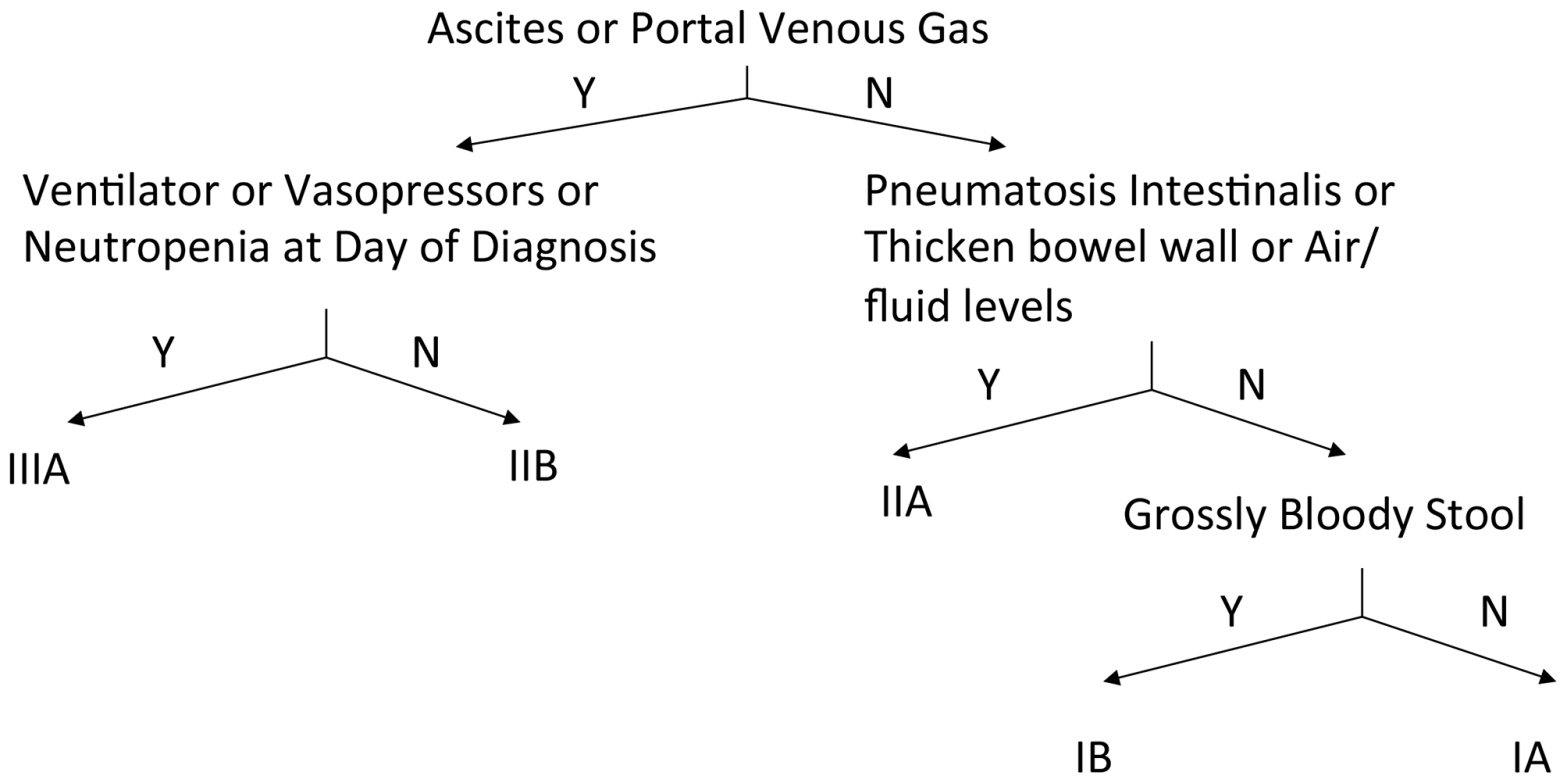
A decision tree to guide the manual assignment of the modified Bell’s staging criteria to the study subjects.

This was a multi-institutional, multi-year study with prospective data collection performed from December 2003 to June 2011 by trained personnel at each participating institution. The time between study eligibility (i.e. clinical concern for NEC or at least Bell’s stage I criteria) and declaration of confirmed NEC (non-progressive or medical) was recorded. Confirmed NEC in the Medical NEC cohort was defined as the presence of pneumatosis. Confirmation of Surgical NEC was defined at the time of surgery. The time from initial clinical suspicion to the progression to surgical disease averaged 2 days. This implies that a prognostic tool implemented at the time of initial clinical suspicion would potentially allow for the timely implementation of new or experimental management strategies. Informed consent was obtained from the parents of all enrolled subjects. This study was approved by the human subjects protection programs at each participating institute of the NEC consortium. The institutional review boards (IRBs) are the Institutional Review Board for Human Subject Research for Baylor College of Medicine and Affiliated Hospitals (BCM IRB), the Children’s Hospital of Philadelphia Institutional Review Board, the Johns Hopkins Medicine Institutional Review Boards, Nationwide Children’s Hospital Institutional Review Board, and the Human Research Protection Program. A binary decision tree (Figure 1) was constructed to post hoc classify the NEC consortium subjects into different stages according to Bell’s staging criteria. Accordingly, all subjects in the NEC database were manually assigned to a specific Bell’s stage according to the binary tree provided. Sixty-seven out of the 587 subjects were classified as Bell’s stage IIIb (pneumoperitoneum). Since these subjects could represent another commonly acquired neonatal intestinal disease (spontaneous intestinal perforations, SIP), they were excluded from further analysis. De-identified clinical and laboratory data were extracted for multivariate analysis.

All subjects were randomized (R random sampling method) into cohorts for statistical training (2/3) and testing (1/3) analyses by categories of Bell’s stages I, II, III (Table 1, training N = 343; testing N = 177). Demographic data was analyzed using R (http://www.r-project.org/) epicalc package [8]. Chi-square test, Fisher's exact test and Kruskal-Wallis test were applied. All subjects’ significant clinical datasets (27 parameters) were extracted and later used in the statistical learning to develop NEC staging and outcome scoring metrics. Significant historic factors include: “Feeding intolerance”, “Apneic/bradycardic episode”, “Oxygen desaturation episode”, “Grossly bloody stools”. Physical exam parameters included: “Abdominal distentions”, “Capillary refill time (CRT) greater than 2 seconds”, “Abdominal wall discoloration”, “Abdominal tenderness”. Medical history parameters included: “Was on a ventilator on the day he/she met the protocol definition of NEC”, “Was on vasopressors on the day he/she met protocol definition of NEC”. Radiographic findings include: “Pneumatosis intestinalis”, “Portal venous gas”, “Ileus”, “Dilated bowel”, “Air/fluid levels”, “Thickened bowel walls”. Clinical laboratory data included: WBC (×10^3^/mm^3^); Neutrophils (%), ANC (absolute neutrophils count) (×10^9^/L), bands (%), Band count (×10^9^/L), Platelets (×10^3^/uL), Bicarbonate (meg/L), pH value, pH site and “Abdominal pain”. Additional parameters of significance included acidosis when pH value < 7.3 and pH site is arterial or when bicarbonate value < 16, neutropenia when neutrophil count < 10^9^/L, and thrombocytopenia when platelet count < 100,000 per microliter.

**Table 1:** Demographics of NEC patients by Bell’s staging criteria.

	Training				Testing			
	Stage I	Stage II	Stage III	*p*-value	Stage I	Stage II	Stage III	*p*-value
	N = 182 (53.1%)	N = 132 (38.5%)	N = 29(8.5%)		N = 92 (52.0%)	N = 66 (37.3%)	N = 19 (10.7%)	
Male†	96 (52.7%)	82 (62.1%)	18 (62.1%)	0.217	52 (56.5%)	33 (50.0%)	14 (73.7%)	0.184
Race‡				0.023				0.037
White	88 (48.4%)	84 (63.6%)	9 (31.0%)		36 (39.1%)	33 (50.0%)	5 (26.3%)	
African American	60 (33.0%)	28 (21.2%)	12 (41.4%)		31 (33.7%)	18 (27.3%)	11 (57.9%)	
Asian	6 (3.3%)	3 (3.0%)	0 (0%)		5 (5.4%)	0 (0%)	0 (0%)	
Native Hawaiian or	0 (0%)	0 (0%)	0 (0%)		0 (0%)	1 (1.5%)	2 (10.5%)	
Pacific Islander								
American Indian or	0 (0%)	0 (0%)	0 (0%)		1 (1.1%)	2 (3.0%)	0 (0%)	
Alaskan Native								
Unknown	23 (12.6%)	12 (9.1%)	6 (20.7%)		17 (18.5%)	10 (15.2%)	1 (5.3%)	
Other	5 (2.7%)	4 (3.0%)	2 (6.9%)		2 (2.2%)	2 (3.0%)	0 (0%)	
Gestational Age (weeks)§	28 (26,31)	31 (27,34)	30 (27,31)	<0.001	28 (26,31)	30.5 (27,33.8)	28 (26.5,29)	0.028
Birth Weight (grams)§	941 (748.5,1437.5)	1402 (937.2,2000.8)	1110 (910,1540)	<0.001	931 (749.8,1428.8)	1317.5 (830,1746.2)	1028 (875,1256.5)	0.063
Birth Length [22] §	35 (31.2,40)	38.5 (33,43)	37 (32,39)	0.004	34.7 (32.5,40.1)	38 (33,42)	36 (33.8,38.8)	0.249
Birth Head Circumference [22] §	24.6 (22.6,27.5)	27.5 (23.2,30.5)	26 (22.5,27.5)	<0.001	25.1 (23.2,28.5)	26.8 (23.5,29.5)	24.5 (23.1,26.8)	0.173
Medical/Surgical NEC‡				<0.001				<0.001
Medical	134 (73.6%)	86 (65.2%)	6 (20.7%)		73 (79.3%)	44 (66.7%)	1 (5.3%)	
Surgical	30 (16.5%)	40 (30.3%)	20 (69.0%)		15 (16.3%)	18 (27.3%)	17 (89.5%)	
Missing	18 (9.9%)	6 (4.5%)	3 (10.3%)		4 (4.3%)	4 (6.1%)	1 (5.3%)	

† Chi-square test is used. N is reported with percentages in parentheses.

‡ Fisher's exact test is used. N is reported with percentages in parentheses.

§ Kruskal-Wallis test is used. Median is reported with IQR in parentheses.

### Development of the NEC staging scoring metric

We performed the generalized linear mixed-effects models (GLMMs) [9] model analysis using R library function glmer from lme4 package [10] utilizing the 27 clinical parameters at each subject’s initial clinical presentation. The NEC staging discriminant function is the GLMM, and the method has the form response ∼ terms where response is the ordinal factor (stage I, II, III) and terms are the 27 or subset of the 27 clinical parameters. Random effects of different subject hospital sites were considered during the training process of the GLMMs model. Regression coefficients for each of the variables along with the standard errors and *t*-values were computed. We also performed a Wald-test to evaluate the overall model fit using the R “lmtest” package [11]. Subsequently we reran the GLMMs model with the robust standard errors using R package “sandwich” [12] to adjust for heterogeneity in the model. This analysis yielded similar significant results, but gave more realistic *p*-values. 9 of the original 27 parameters were identified as significant and selected to construct the linear regression model. This 9-parameter subset included: “Abdominal pain”, “Pneumatosis intestinalis”, “Portal venous gas”, “Dilated bowel”, “Air/fluid levels”, “Thickened bowel walls”, “WBC (×10^3^/mm^3^) ”, “Neutrophils (%)”, and “Neutrophil count”. The computed value of the response variable as well as the “output” of the model was defined as the NEC staging score. Finally, we used the “predict” function to obtain the predicted values for the blind testing data set.

### Development of the NEC risk of disease progression metric

36 patients were removed from the dataset (N = 520) due to incomplete data and inability to determine medical or surgical outcomes. Linear discriminant analysis (LDA) [13] was applied to stratify individual subjects based on clinical data. Specifically R library MASS [14] function “lda” was utilized. Coefficients of linear discriminants (LD1) were calculated as a measure of the association of each variable with the final diagnosis. LDA created linear combinations of these clinical variables and calculated coefficients LD1 to optimize separation between medical NEC and surgical NEC groups. The NEC outcome discriminant function is the LDA, and the predictive method has the form response ∼ terms where response is the factor (progressive and non-progressive NEC) and terms are the 27 or subset of the 27 clinical parameters. To gauge the performance of the NEC outcome LDA model to classify non-progressive from progressive NEC subjects, we performed receiver operating characteristic (ROC) analyses [15].

The LDA discriminant scoring metric, designated as the NEC outcome score, enables the clinical variables to be collectively interpreted on a scale, rather than a strict binary discrimination. We calculated NEC outcome scores based on clinical findings (27 parameters) for all subjects in the training cohort and stratified them into subgroups with low, intermediate, and high scores based on 90% correct classification in the low (10% probability of medical NEC) and high risk (90% probability of surgical NEC) groups.

## Results

### Demographics

In both training and testing data sets (Table 1), race, gestational age, birth weight, length and head circumference related differences were observed between different subjects according to their Bell’s stage. Statistical differences among Bell’s stage I, II and III NEC were observed in race (*p*-value: training 0.023 and testing 0.037), gestation age (*p*-value: training < 0.001 and testing 0.028), birth weight (*p*-value: training < 0.001 and testing 0.063), birth length (*p*-value: training 0.004 and testing 0.249), and birth head circumference (*p*-value: training < 0.001 and testing 0.173). No statistical differences were observed in gender among the different NEC stages.

For the risk of disease progression model, there were differences in gender, gestational age, birth weight, length and head circumference between different subjects according to their progressive/non-progressive designation and outcome. In progressive NEC, there were slightly more male infants (*p*-value: training 0.068 and testing 0.024), smaller gestational age (*p*-value: training 0.067 and testing 0.037), smaller birth weight (*p*-value: training 0.09 and testing 0.095), smaller birth length (*p*-value: training 0.132 and testing 0.033), and smaller birth head circumference (*p*-value: training 0.002 and testing 0.005). No statistical differences were observed for race between progressive and non-progressive NEC.

### Automation of the NEC Bell’s Staging Assignment

**Figure 2:**
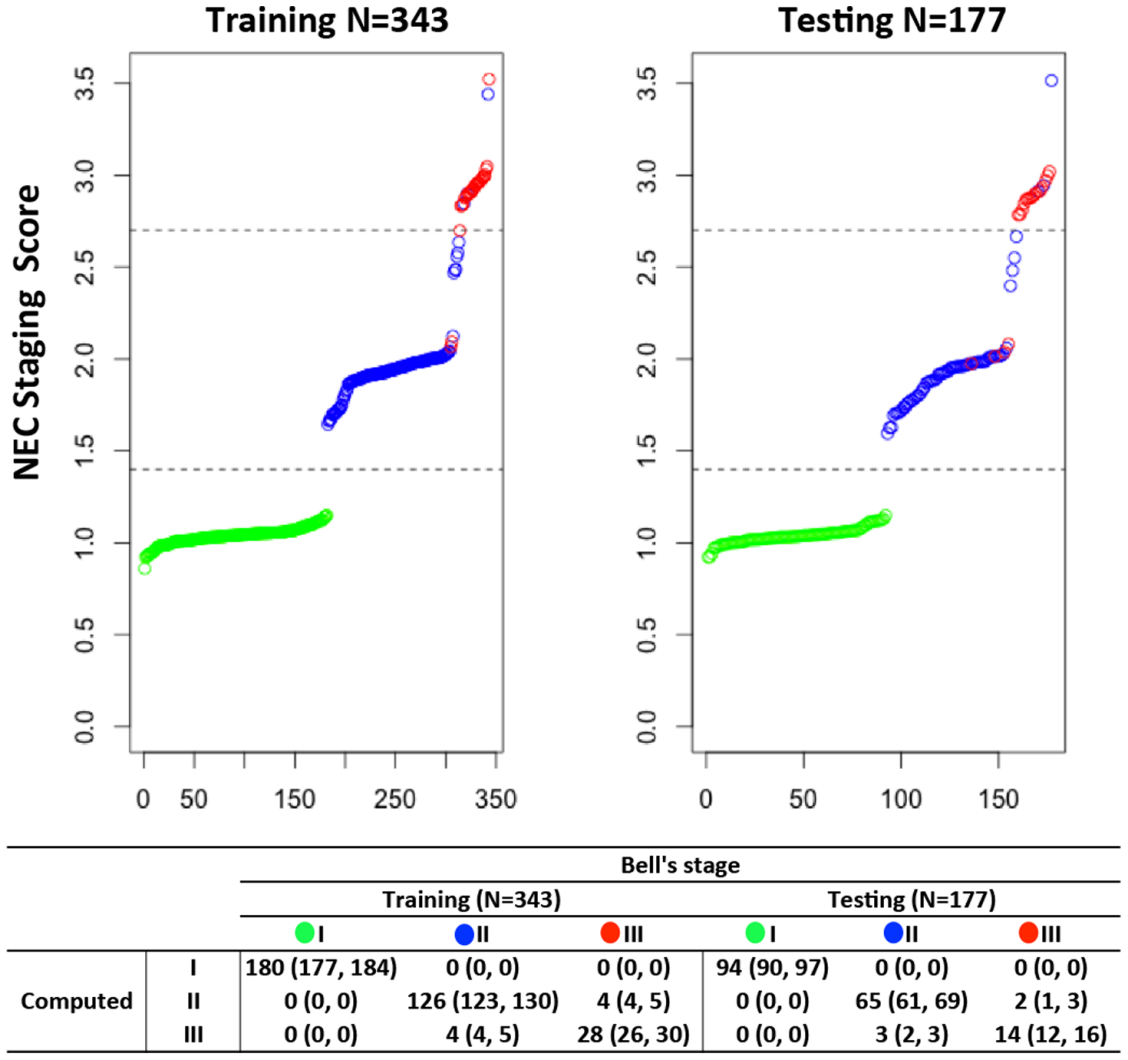
Automated NEC staging assignment results. Left: modeling training. Right: blind testing. Bottom: manual versus automated NEC staging assignment comparative analysis. To gauge the impact of different training/testing cohort partition on the statistical learning, we performed a bootstrapping analysis that randomly partitioned the cohorts into 100 different training/testing sets. Results were summarized where median and interquartile range (IQR) values were calculated for each comparative category.

Recognizing that Bell’s stage requires significant clinician input to accurately classify patients as was done in Figure 1, we initially sought to create a model that could diagnose NEC according to presenting stage without the need for manual assignment. This effort was further motivated by a desire to remove the subjective nature of current NEC diagnostic algorithms and as a prelude to automating these tasks to accommodate real-time computation. We hypothesized that an automated staging system similar to the Bell’s staging criteria could be developed using clinical data at the time of presentation. Utilizing a 9-parameter model (Figure 2), subjects were objectively assigned a stage at the time of presentation thus avoiding the need for manual assignment. The, horizontal lines in Figure 2 designate the scores where 90% of stage I (or stage III) subjects were classified correctly and matched the original manual Bell’s stage designation (Figure 1) of the training subjects. The computed NEC stage demonstrated promising agreement with the clinical categorical assignment: Stage I (training: 100%, testing: 100%), Stage II (training: 97.7%, testing: 94.0%), Stage III (training: 89.7%, testing: 83.3%). In both training and testing, none of the Stage I or III patients was mistakenly cross-assigned to Stage III or I respectively.

### Development of NEC outcome scoring metrics

**Figure 3:**
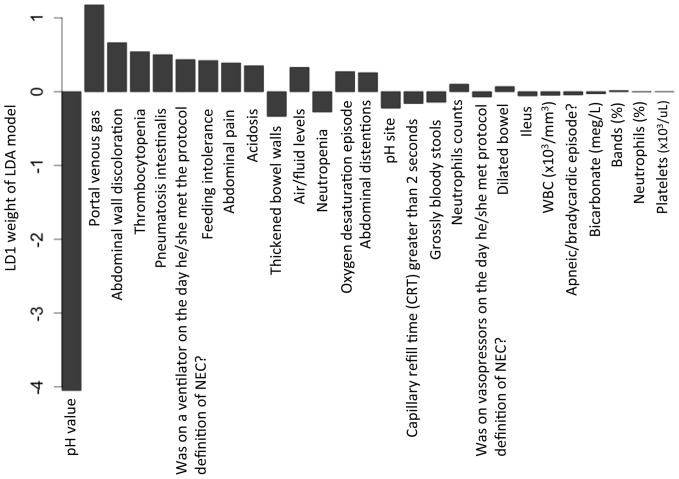
Clinical variable’s contribution (LD1) to the NEC outcome LDA model. LDA: Linear discriminant analysis. LD1: first discriminant variable.

**Figure 4:**
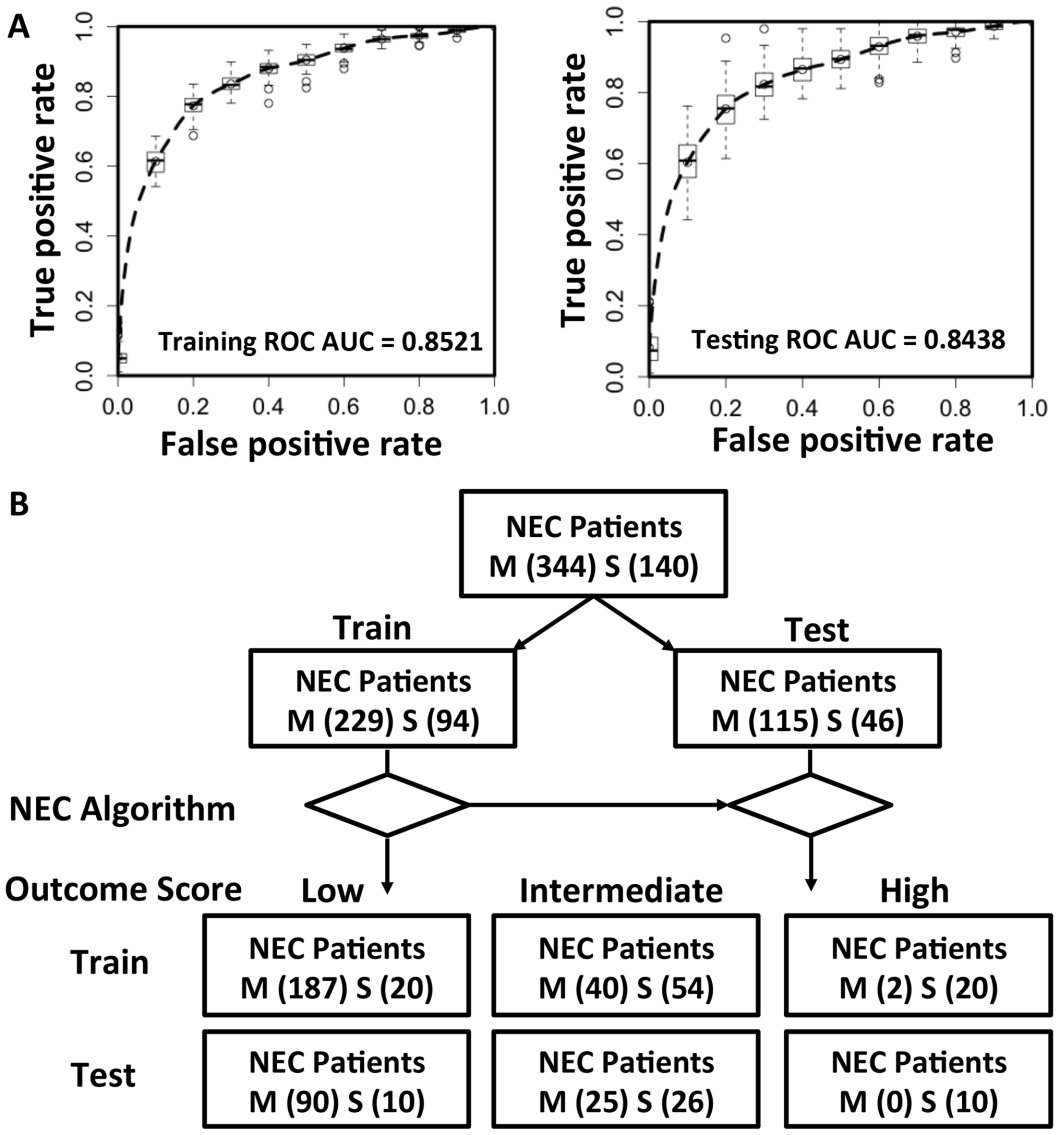
NEC outcome predictive results. A. ROC AUC analysis. To gauge the impact of different training/testing cohort partition on the statistical learning, we performed a bootstrapping analysis that randomly partitioned the cohorts into 100 different training/testing sets. The distribution of 100 ROC curves, training and testing respectively, are illustrated. B. Use of the NEC outcome prediction metric to risk-stratify NEC subjects into low, intermediate and high risk groups.

In order to determine whether statistical modeling could be used to help predict patient outcome, a strategy of risk stratification rather than binary prediction was pursued. An LDA based model (Figure 3) was used to risk stratify the training and testing subjects into 3 levels of risk for progression of NEC (Figure 4 and Figure S2): low (score < 0.1313; in training cohort:187 medical NEC and 20 surgical NEC; in testing cohort: 90 medical NEC and 10 surgical NEC testing subjects), intermediate 0.1313≤ score ≤ 2.2160; in training cohort: 40 medical NEC and 54 surgical NEC; in testing cohort: 25 medical NEC and 26 surgical NEC) and high (score > 2.2160; in training cohort: 2 medical NEC and 20 surgical NEC; in testing cohort: 0 medical NEC and 10 surgical NEC). The intermediate groups include 29.1% and 31.7% of the total training and testing subjects respectively whose outcome could not be confidently predicted by our clinical scoring algorithm. ROC AUCs of the clinical score for a determination of risk of NEC progression were 0.84 and 0.85 in training and testing cohorts respectively. With this NEC outcome scoring metric, we can effectively stratify the NEC subjects according to their NEC progressive risk using criteria obtainable at the time of disease presentation, resulting in 80.5% of the medical subjects (N = 277) assigned with 10% risk of being progressive, and 21.4% of the surgical NECs (N = 30) assigned with 90% risk of being progressive. Overall, although our predictive method yielded encouraging positive predictive rates (PPV) of 90.2% and 93.8% for accurate (90% confidence interval) medical and surgical NEC outcome prediction respectively, there were still 18.9% medical NEC and 57.1% surgical NEC subjects that remained undetermined, and 0.6% of medical NEC and 21.4% surgical NEC subjects in-correctly predicted.

**Figure 5:**
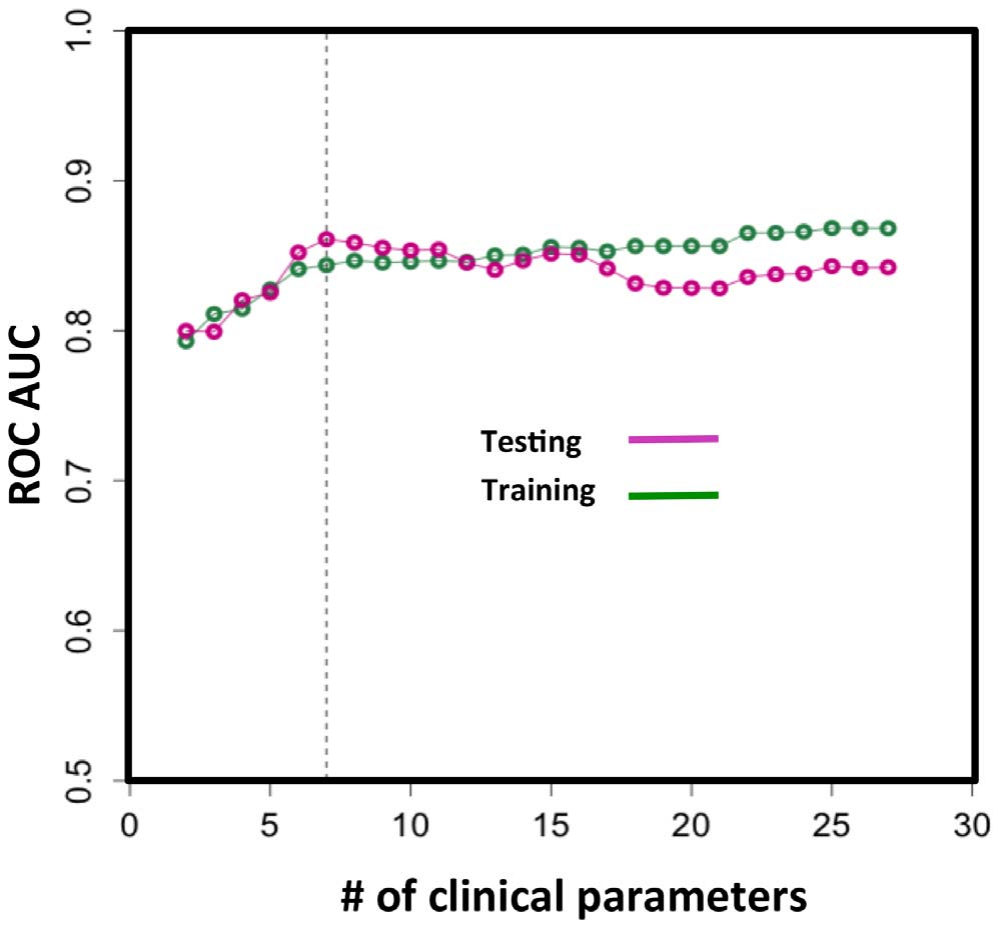
NEC outcome predictive LDA models with reduced number of variables (listed in descending order from right to left in Figure 3 by the absolute value of their weights). The model performance was gauged by ROC analysis. Vertical dotted line: the model performance deteriorates when the model’s panel size is less than 7 parameters.

Next, we set to explore the impact on NEC outcome prediction incurred by a reduction in the number of clinical parameters available for risk stratifying. We reasoned that many potential patients would have incomplete data in the early presentation or suspicion of disease. The practical consideration is that when information of a particular clinical parameter is unavailable, the algorithm can still provide a useful determination of risk. Beginning with the smallest-weighted parameter (Figure 3, right to the left), we iteratively reduced the number of parameters to construct outcome prediction models of increasingly smaller panel sizes. The models were developed using training data and tested blindly on the testing dataset. AUCs were computed and ROC curves plotted (Figure 5). AUC analyses indicated that the model performance deteriorates when the model’s panel size is less than 7 parameters.

## Discussion

The clinical presentation of neonates with signs and symptoms suggestive of NEC can be obscured by similarities to other inflammatory conditions of the newborn. Moreover, there are no reliable prognostic signs of progressive NEC that herald disease progression prior to patients reaching an irreversible state requiring surgery. Clinicians have therefore utilized combinations of clinical indicators and experience to guide patient management. Specifically, NEC is diagnosed through a combination of clinical, radiographic and laboratory findings that in aggregate define the original Bell’s staging criteria. We automated a NEC stage assignment at diagnosis according to a 9-feature panel with utility comparable to the manual assignment of Bell’s staging criteria. Overall there was generally good agreement between the computed and manually assigned stages for the lower stage groups (100% testing) upon presentation. The correlation between the computed and manual stage deteriorated with increasing stage, however (Stage II 94.0% and Stage III 83.3% testing). Although Bell’s staging assignment at the time of initial diagnosis is useful, it is a static diagnostic algorithm that was not intended to forecast disease progression. Our data (Figure S3) reveal that the majority of patients that develop progressive NEC do not present with a fulminate course and would therefore benefit from a better prediction model.

Bell’s original staging criteria and subsequent modifications have been used by clinicians to apply diagnostic labels to patients as being either “suspicious”, “confirmed” or representing “advanced stage” NEC. Additional follow-up measurements, including frequent abdominal radiographs and laboratory values are obtained in an attempt to define further patient trajectory of disease. A label of confirmed NEC normally requires a finding of pneumatosis intestinale on an abdominal radiograph in an infant that has other systemic signs and symptoms suggestive of illness. Although pneumatosis is diagnostically definitive, it does not predict outcome or suggest a specific therapy. The label of “advanced NEC” implies that the disease has progressed to a point of irreversible intestinal injury including intestinal necrosis producing perforation and thus requiring an operation to excise the affected intestine. Unfortunately, the designation of progressive NEC by utilizing hard clinical signs only occurs at a time when the disease has already advanced and there remains little opportunity to offer novel treatment options that might alter the course of disease.

The definition of NEC was important for this study and was implemented in such a way that our datasets did not contain infants with potential SIP. SIP is a separate and distinct disease entity. In many national datasets, the surgical NEC subsets are as much as 20% SIP, and in our original database there was ambiguity in the diagnosis of 67/587 infants given the initial presentation of pneumoperitoneum. Not modeling to predict NEC associated death is a limitation of the study, which clearly should be addressed in the future when more comprehensive datasets are available. There were 75 deaths amongst NEC subjects across the five children’s hospitals in our database between 2003 and 2010. Unfortunately among these subjects, most of the needed clinical parameters were not collected.

Utilizing this curated large multicenter prospective clinical database, we sought to develop a prediction model for the risk of NEC progression. We reasoned that NEC risk stratification would find the most clinical utility in patients presenting without an obvious clinical trajectory. Initially, in order to build a risk of disease progression model we utilized 27 available data points recorded at the time of initial clinical suspicion of NEC. These data points were used to construct a tiered risk stratification scheme in order to increase its flexibility and improve upon the binary (or categorical) classification suggested by Bell’s staging criteria (i.e. advanced disease or something less). Moreover, a majority of subjects with low risk disease (80.5%) can be positively identified with high confidence (90% CI) while only a small percentage (21.4%) of subjects that were found to have eventual progressive disease could be identified *a priori* and at the time of initial presentation with a high degree of confidence (90% CI). Thus, despite a good positive predictive value for the very low and high-risk groups identified by this algorithm, there remains 18.9% of the medical and 57.1% of the surgical subjects that are indeterminate and would require further testing.

Not surprisingly, the previously described NEC parameters of metabolic acidosis (pH) [16]–[19] and portal venous gas (PVG) [16]–[18] were found by our multivariate machine learning to be the most weighted predictors (Figure 3) on the NEC outcome score. This congruency suggests that our machine learning methodology identified both statistically and pathophysiologically significant predictors of NEC. We speculate that this type of risk stratification will provide several relevant clinical applications to be determined in future studies for either identifying patients for additional diagnostic/prognostic testing or perhaps alternative therapies (i.e. earlier surgery). Despite the simplicity of design, this type of risk stratification with an AUC of 0.85 testing by ROC analyses was superior to Bell’s staging criteria in projecting patient outcomes with NEC. Still, there remained a full 30.0% of patients found to be of intermediate risk that would require additional testing or a novel test to help further define these patients’ risk of progression. This is one of the principle limitations of our study that we posit can only be resolved through the discovery and addition of additional features including possible molecular biomarkers to the model. Due to this limitation, caution should be applied in using our proposed risk stratification model to guide management of infants with NEC. Our work suggests future NEC outcome biomarker studies should focus on the discovery of markers that can differentiate NEC subjects with intermediate risk scores for progression. We speculate that biomarker testing that is in development by several groups including our own may address this unmet need [20], [21]. It also remains to be determined whether iterative longitudinal testing of patients with an obscure clinical course might be further instructive using the current clinical parameter model.

In order to facilitate further clinical validation, we have also sought to determine the performance of the progression risk model using incomplete data. We reasoned that in the clinical setting many patients would be unlikely to have complete datasets to fulfill model requirements and achieve risk stratification. Our iterative testing and ROC curve analysis demonstrated that the model maintains robust performance characteristics over a wide range of values. This is encouraging and highlights an additional advantage of this algorithm given its ability to accommodate missing values while still providing a useful output. By comparing a new patient to all of the patient data points in the database one can determine where the new patient clusters in an unsupervised analysis.

A challenge to deriving predictive algorithms is to show utility relative to the existing status quo. As any predictive model will be most useful if able to reveal an obscure disease or forecast an unforeseen event, our models hold significant potential for clinical utility relative to currently employed methods of NEC diagnosis and prognosis. With ongoing refinement, these algorithms may accurately identify low-risk patients and perhaps enable implementation of novel therapeutic approaches for high-risk patients as they are developed.

## Supporting Information

Figure S1 Point-of-care applications of proposed NEC algorithm. To allow point of care utilization of our innovative NEC algorithm, we designed and created a NEC analytical website application at http://translationalmedicine.stanford.edu/cgi-bin/NEC/nec.pl to permit remote diagnostic and prognostic guidance, based on patient demographics, historic factors, physical exam, medical history, fluid intake and nutrition, radiographic findings, laboratory tests, and other findings. The simplified web application was built and hosted at: http://translationalmedicine.stanford.edu/cgi-bin/NEC/index.pl, which is based on the aforementioned feature selection result. In addition, a smartphone application was developed to support point of care NEC management through the utilization of NEC clinical database and algorithms. Implementation technical details: with a Model View Controller (MVC) design, we applied a previously developed server-based bio-computational framework [23] to allow physicians remote access to our NEC algorithms. The algorithm and associated web application were implemented using R and PERL (http://www.perl.org/) respectively. The iPhone application was developed in the integrated development environment [24] Xcode 4.6.2 and iOS SDK 6.1.2 using Objective C. Later it was tested using iPhone Simulator 6.1, iPhone 5 and new iPad, it was supported by iOS 6.1 or higher versions. Recognizing the need for automation to facilitate adoption and to potentially shield clinicians from the complex details and computation required to risk stratify patients using these algorithms, we created server-based applications that are capable of providing immediate risk stratification via a graphical output to users. We speculate that this type of mobile application and server-based computation will facilitate robust validation and additional longitudinal testing of the algorithms provided. We also submit that this type of electronic application will promote adoption of the existing algorithms while promoting the evolution and improved performance of the existing prediction models through the addition of additional subjects data.
(TIF)

Figure S2 Results of the risk stratification analysis for the progression of NEC.
(TIF)

Figure S3 Exploratory correlation analysis between NEC outcome and the assigned Bell’s staging determined at initial clinical presentation. A. Density plots of Bell’s stage I, II, III patients relative to NEC outcome (medical, surgical NEC). The density plot of Bell’s stage I-III subjects was analyzed in terms of NEC outcomes (medical, surgical). The medical NEC patient number negatively and the surgical NEC patient number positively correlated with the increment of Bell’s stage: 32.1%, 41.4% and 26.4% of the progressive (surgical) NEC subjects were in NEC staging I, II and III while 52.1%, 38.8% and 9.1% of all NEC subjects were in NEC staging I, II and III respectively. Among all stage III patients, 84.1% were ultimately progressive NEC and had subsequent surgery. Assuming all stage III patients are progressive, the positive predictive value is 84.1% and negative predictive value is 76.6%. However, this algorithm failed to predict the progressive outcome of 73.6% of surgical NEC patients at the time of diagnosis (N = 45 in Stage I, N = 58 in Stage II). B. Comparative ROC curve analysis using either manual Bell’s staging criteria or the presented automated Bell’s staging assignment to forecast NEC outcome.
(TIF)

## References

[1] Guthrie SO, Gordon PV, Thomas V, Thorp JA, Peabody J, et al (2003) Necrotizing enterocolitis among neonates in the United States. Journal of perinatology 23: 278–285. 12774133 10.1038/sj.jp.7210892

[2] Kamitsuka MD, Horton MK, Williams MA (2000) The incidence of necrotizing enterocolitis after introducing standardized feeding schedules for infants between 1250 and 2500 grams and less than 35 weeks of gestation. Pediatrics 105: 379–384. 10654959 10.1542/peds.105.2.379

[3] Lemons JA, Bauer CR, Oh W, Korones SB, Papile L-A, et al (2001) Very low birth weight outcomes of the National Institute of Child Health and Human Development neonatal research network, January 1995 through December 1996. Pediatrics 107: e1–e1. 11134465 10.1542/peds.107.1.e1

[4] Blakely ML, Lally KP, McDonald S, Brown RL, Barnhart DC, et al (2005) Postoperative outcomes of extremely low birth-weight infants with necrotizing enterocolitis or isolated intestinal perforation: a prospective cohort study by the NICHD Neonatal Research Network. Annals of surgery 241: 984. 15912048 10.1097/01.sla.0000164181.67862.7f PMC1359076

[5] Blakely ML, Tyson JE, Lally KP, McDonald S, Stoll BJ, et al (2006) Laparotomy versus peritoneal drainage for necrotizing enterocolitis or isolated intestinal perforation in extremely low birth weight infants: outcomes through 18 months adjusted age. Pediatrics 117: e680–e687. 16549503 10.1542/peds.2005-1273

[6] Hintz SR, Kendrick DE, Stoll BJ, Vohr BR, Fanaroff AA, et al (2005) Neurodevelopmental and growth outcomes of extremely low birth weight infants after necrotizing enterocolitis. Pediatrics 115: 696–703. 15741374 10.1542/peds.2004-0569

[7] Bell MJ, Ternberg JL, Feigin RD, Keating JP, Marshall R, et al (1978) Neonatal necrotizing enterocolitis. Therapeutic decisions based upon clinical staging. Annals of surgery 187: 1. 413500 10.1097/00000658-197801000-00001 PMC1396409

[8] Chongsuvivatwong V (2011) epicalc: Epidemiological calculator. R package version 2 (13): 2.2.

[9] Rabe-Hesketh S, Skrondal A (2008) Generalized linear mixed-effects models. Longitudinal data analysis: 79.

[10] Bates D, Maechler M, Dai B (2008) The lme4 package. Computer software manual retrieved from http://cran.r-project.org/web/packages/lme4/lme4.pdf. Accessed 2014 Jan 31

[11] Zeileis A, Hothorn T (2002) Diagnostic checking in regression relationships. R news 2: 7–10.

[12] Zeileis A (2004) Econometric computing with HC and HAC covariance matrix estimators.

[13] Balakrishnama S, Ganapathiraju A (1998) Linear discriminant analysis-a brief tutorial. Institute for Signal and information Processing.

[14] Ripley BD (2002) Modern applied statistics with S: Springer.

[15] Zweig MH, Campbell G (1993) Receiver-operating characteristic (ROC) plots: a fundamental evaluation tool in clinical medicine. Clinical chemistry 39: 561–577. 8472349

[16] Moss RL, Kalish LA, Duggan C, Johnston P, Brandt ML, et al (2008) Clinical parameters do not adequately predict outcome in necrotizing enterocolitis: a multi-institutional study. J Perinatol 28: 665–674. 18784730 10.1038/jp.2008.119

[17] McCormack CJ, Emmens RW, Putnam TC (1987) Evaluation of factors in high risk neonatal necrotizing enterocolitis. J Pediatr Surg 22: 488–491. 3112356 10.1016/s0022-3468(87)80202-6

[18] Bell MJ, Ternberg JL, Feigin RD, Keating JP, Marshall R, et al (1978) Neonatal necrotizing enterocolitis. Therapeutic decisions based upon clinical staging. Ann Surg 187: 1–7. 413500 10.1097/00000658-197801000-00001 PMC1396409

[19] Ballance WA, Dahms BB, Shenker N, Kliegman RM (1990) Pathology of neonatal necrotizing enterocolitis: a ten-year experience. J Pediatr 117: S6–13. 2362230 10.1016/s0022-3476(05)81124-2

[20] Sylvester KG, Ling XB, Liu GY, Kastenberg ZJ, Ji J, et al. (2013) A novel urine peptide biomarker-based algorithm for the prognosis of necrotising enterocolitis in human infants. Gut. 10.1136/gutjnl-2013-305130 PMC4161026 24048736

[21] Sylvester KG, Ling XB, Liu GY-G, Kastenberg ZJ, Ji J, et al. (2014) Urine Protein Biomarkers for the Diagnosis and Prognosis of Necrotizing Enterocolitis in Infants. The Journal of pediatrics. 10.1016/j.jpeds.2013.10.091 PMC4161235 24433829

[22] Bishop C, Tipping M (2009) Variational relevance vector machines; 2000. Citeseer. pp. 46–53.

[23] Ling XB, Cohen H, Jin J, Lau I, Schilling J (2009) FDR made easy in differential feature discovery and correlation analyses. Bioinformatics 25: 1461–1462. 19376824 10.1093/bioinformatics/btp176

[24] Sutherland SM, Ji J, Sheikhi FH, Widen E, Tian L, et al (2013) AKI in hospitalized children: epidemiology and clinical associations in a national cohort. Clin J Am Soc Nephrol 8: 1661–1669. 23833312 10.2215/CJN.00270113 PMC3789331

