# Supplementary figures and images for: A Data-Driven Algorithm Integrating Clinical and Laboratory Features for the Diagnosis and Prognosis of Necrotizing Enterocolitis
Source: PLoS One. 2014 Feb 28;9(2):e89860. doi: 10.1371/journal.pone.0089860 (PMC3938509; doi:10.1371/journal.pone.0089860)

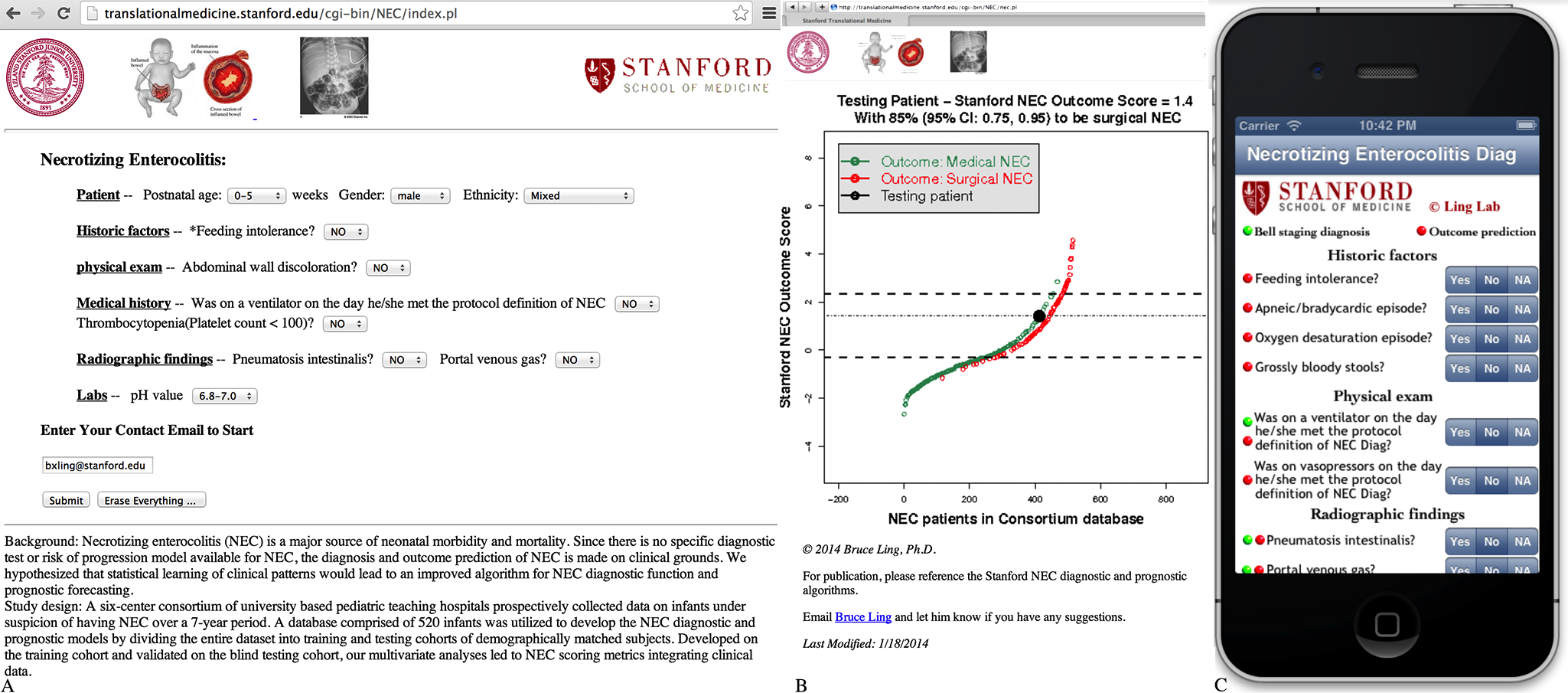

Supplement: Figure S1 — Point-of-care applications of proposed NEC algorithm. To allow point of care utilization of our innovative NEC algorithm, we designed and created a NEC analytical website application at http://translationalmedicine.stanford.edu/cgi-bin/NEC/nec.pl to permit remote diagnostic and prognostic guidance, based on patient demographics, historic factors, physical exam, medical history, fluid intake and nutrition, radiographic findings, laboratory tests, and other findings. The simplified web application was built and hosted at: http://translationalmedicine.stanford.edu/cgi-bin/NEC/index.pl, which is based on the aforementioned feature selection result. In addition, a smartphone application was developed to support point of care NEC management through the utilization of NEC clinical database and algorithms. Implementation technical details: with a Model View Controller (MVC) design, we applied a previously developed server-based bio-computational framework [23] to allow physicians remote access to our NEC algorithms. The algorithm and associated web application were implemented using R and PERL (http://www.perl.org/) respectively. The iPhone application was developed in the integrated development environment [24] Xcode 4.6.2 and iOS SDK 6.1.2 using Objective C. Later it was tested using iPhone Simulator 6.1, iPhone 5 and new iPad, it was supported by iOS 6.1 or higher versions. Recognizing the need for automation to facilitate adoption and to potentially shield clinicians from the complex details and computation required to risk stratify patients using these algorithms, we created server-based applications that are capable of providing immediate risk stratification via a graphical output to users. We speculate that this type of mobile application and server-based computation will facilitate robust validation and additional longitudinal testing of the algorithms provided. We also submit that this type of electronic application will promote adoption of the existing algorithms [file pone.0089860.s001.tif]

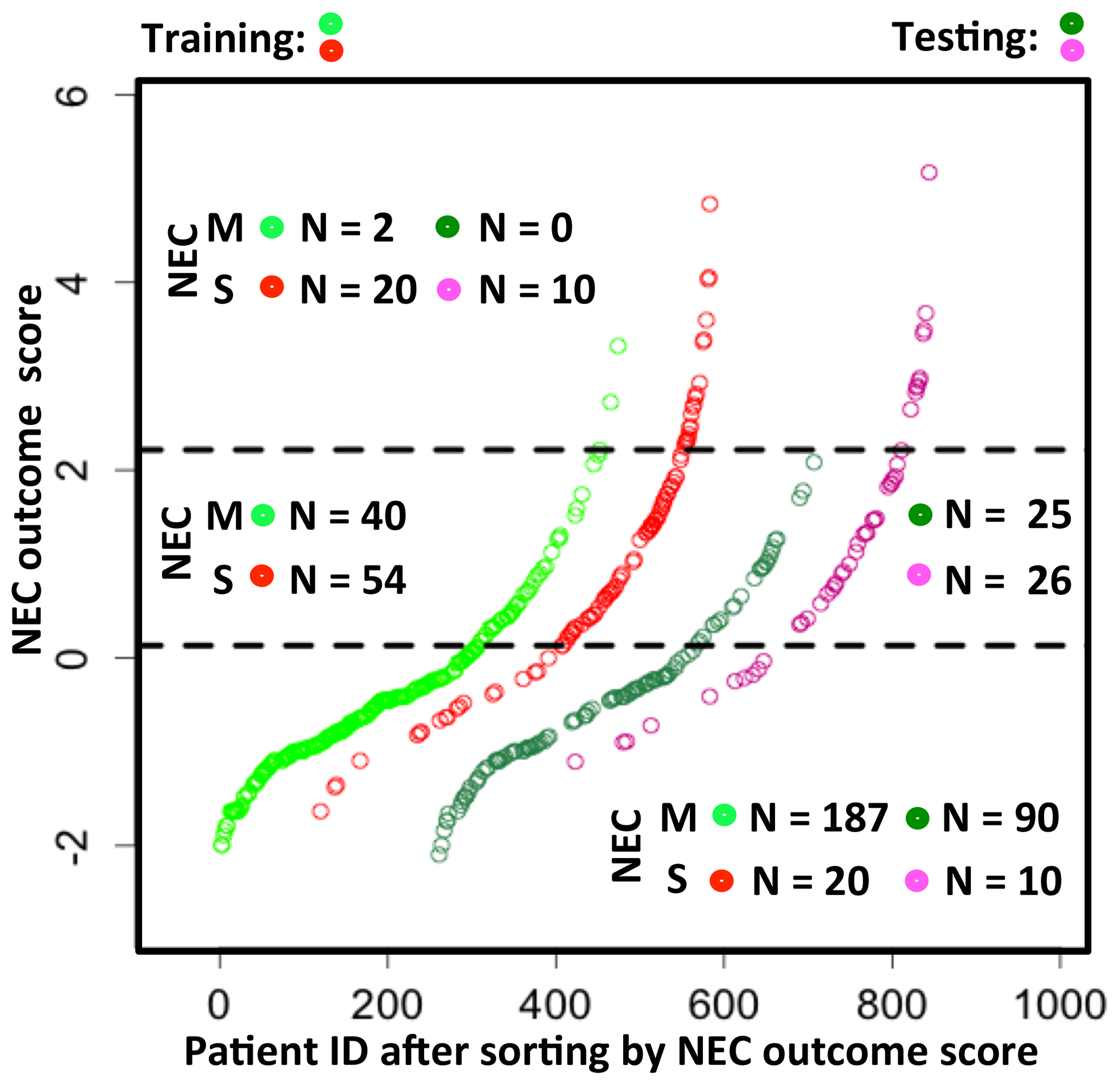

Supplement: Figure S2 — Results of the risk stratification analysis for the progression of NEC. (TIF) [file pone.0089860.s002.tif]

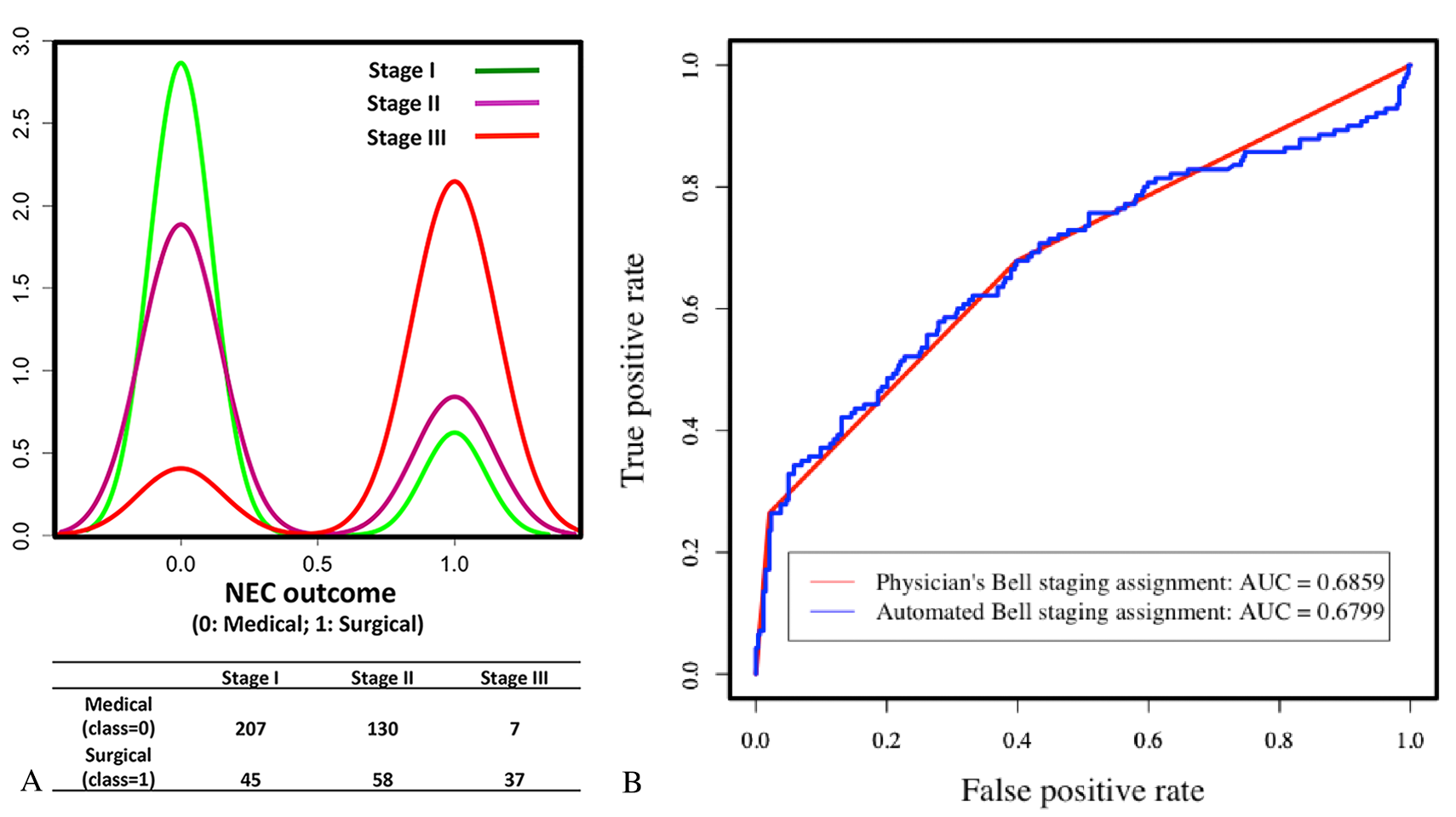

Supplement: Figure S3 — Exploratory correlation analysis between NEC outcome and the assigned Bell’s staging determined at initial clinical presentation. A. Density plots of Bell’s stage I, II, III patients relative to NEC outcome (medical, surgical NEC). The density plot of Bell’s stage I-III subjects was analyzed in terms of NEC outcomes (medical, surgical). The medical NEC patient number negatively and the surgical NEC patient number positively correlated with the increment of Bell’s stage: 32.1%, 41.4% and 26.4% of the progressive (surgical) NEC subjects were in NEC staging I, II and III while 52.1%, 38.8% and 9.1% of all NEC subjects were in NEC staging I, II and III respectively. Among all stage III patients, 84.1% were ultimately progressive NEC and had subsequent surgery. Assuming all stage III patients are progressive, the positive predictive value is 84.1% and negative predictive value is 76.6%. However, this algorithm failed to predict the progressive outcome of 73.6% of surgical NEC patients at the time of diagnosis (N = 45 in Stage I, N = 58 in Stage II). B. Comparative ROC curve analysis using either manual Bell’s staging criteria or the presented automated Bell’s staging assignment to forecast NEC outcome. (TIF) [file pone.0089860.s003.tif]
